# Comparative transcriptome analysis of differentially expressed genes and pathways in male and female flowers of *Fraxinus mandshurica*

**DOI:** 10.1371/journal.pone.0308013

**Published:** 2024-09-12

**Authors:** Kaifang Zhang, Yuan Cao, Xinyue Guo, Fanqiu Kong, Hongran Sun, Tianzhong Jing, Yaguang Zhan, Fenghui Qi

**Affiliations:** 1 State Key Laboratory of Tree Genetics and Breeding, Northeast Forestry University, Harbin, China; 2 College of Life Science, Northeast Forestry University, Harbin, China; 3 School of Forestry, Northeast Forestry University, Harbin, China; University of Naples Federico II, ITALY

## Abstract

*Fraxinus mandshurica* Rupr. (*F*. *mandshurica*) is a dioecious tree species with important ecological and application values. To delve deeper into the regulatory pathways and genes responsible for male and female flowers in *F*. *mandshurica*, we conducted transcriptome sequencing on male and female flowers at four distinct stages. The analysis revealed that the female database generated 38,319,967 reads while the male database generated 43,320,907 reads, resulting in 2930 differentially expressed genes with 1441 were up-regulated and 1489 down-regulated in males compared to females. Following an analysis of Gene Ontology (GO) and Kyoto Encyclopedia of Genes and Genomes (KEGG), four distinct pathways (hormone signal transduction, energy metabolism, flavonoid biosynthesis, and photoperiod) linked to female and male flowers were identified. Subsequently, qRT-PCR verification revealed that *FmAUX/IAA*, *FmEIN3*, and *FmA-ARR* genes in hormone signal transduction pathway are related to female flower development. Meanwhile, *FmABF* genes in hormone signal transduction pathway, *FmGS* and *FmGDH* genes in energy metabolism pathway, *FmFLS* genes in flavonoid biosynthesis pathway, and *FmCaM*, *FmCRY*, and *FmPKA* genes in photoperiod pathway are related to male flower development. This study was the first to analyze the transcriptome of male and female flowers of *F*. *mandshurica*, providing a reference for the developmental pathways and gene expression levels of male and female plants.

## 1. Introduction

The *F*. *mandshurica* is a member of the Fraxinus genus within the Oleaceae family, one of northeastern China’s three hardwood broadleaf tree families [1]. It is a crucial broad-leaved timber species and a key species in the climax forest community in Northeast China [2, 3]. Additionally, because of the beautiful pattern and hard material features, it has significant economic value as an excellent precious wood in production practice [4]. These unique and valuable characteristics make it essential to study species value and ecological diversity.

The sexual development of higher plants is primarily evident in a specialized organ of flower [5]. *F*. *mandshurica* typically takes 15–20 years of growth before it blooms for the first time, and before that it is difficult to identify gender [6]. *F*. *mandshurica* is a dioecious plant, meaning male and female flowers are present on separate individuals. Dioeciousness is a means to avoid inbreeding and maintain genetic diversity, and it is possible that dioecious plants descended from hermaphroditic ancestors [7, 8]. Female plants are primarily responsible for population reproduction, while male plants are more commonly used for economic growth, reforestation, and timber production [9]. As a result, the study of molecular, genetic [10], and ecological behavior has been extensively conducted on both bisexual reproduction and dioeciousness [11, 12]. Therefore, we conducted a transcriptome analysis study on the male and female flowers of *F*. *mandshurica*.

Recently, RNA-seq has become a popular method for analyzing plant transcriptomes, allowing researchers to identify key pathways and genes [13, 14]. RNA-seq results can be used for various analyses such as de novo transcription assembly [15], transcription factor analysis [16], differential expression detection [17], and gene annotation [18]. While sex chromosomes have not been found in *F*. *mandshurica*, previous transcriptome studies have shown that female and male plants differ greatly in energy metabolism and flavonoid biosynthesis [19, 20]. It has been demonstrated that the plant hormone and photoperiod play a crucial role in flower development [21, 22], for instance, genes such as cytokinin [23], *FLCs* [24], and *PRR37* [25] respectively play important roles in the above process. These differences suggest that sex-specific adaptability to different environments might exist [26]. In addition, research has shown that flowering regulation in *Arabidopsis thaliana* involved at least six pathways, including the photoperiod, vernalization, ambient temperature, autonomous, gibberellin, and age pathways [27].

In previous research, we closely examined the morphology of male and female flowers in *F*. *mandshurica* during various stages of development [28]. To further refine the understanding of the genetic differences between male and female flowers in *F*. *mandshurica*, the genes of transcriptome of male and female flowers were annotated and analyzed. Using these data, four pathways related to flower development were further analyzed as hormone signal transduction pathway, energy metabolism pathway, flavonoid biosynthesis pathway, and photoperiod pathway. Additionally, we compared the expression of genes in the above pathways in adult male and female leaves as well as young leaves to better identify the difference genes in flower development.

## 2. Materials and methods

### 2.1 Plant material

The materials utilized in this study were sourced primarily from the Qingshan forest seed orchard of Weihe Forestry Bureau, located in Shangzhi City, Heilongjiang Province, China. In order to ensure consistency, 30 *F*. *mandshurica* with similar growth conditions in the same provenances were selected as experimental materials, including 15 male and 15 female trees. The male flower contains four cell stages, namely archesporial cell phase, pollen mother cell phase, meiosis cell, and cell of loose pollen phase. Similarly, the female flower has four cell stages: archesporial cell phase, embryo-sac mother cell phase, meiosis cell, binucleated cell [29]. The leaves were collected from adult male and female trees, as well as young trees (2 years old). Samples were manually picked, frozen immediately in liquid N_2_ and stored at -80°C until further use. Three parallel experiments were conducted to ensure the accuracy of the results.

### 2.2 RNA extraction and library preparation

The TaKaRa MiniBEST universal RNA extraction kit (TaKaRa, Tokyo, Japan) was used to extract the total RNA from male and female flowers, and simultaneously remove DNA from RNA. The RNA concentration was measured using a Nanodrop 2000 instrument (Thermo Fisher Scientific, Waltham, MA, USA). To assess the quality of RNA, agarose gel electrophoresis was performed (S1 Fig). Two samples of female and male flowers were prepared by mixing RNA samples from 4 different periods of male and female flowers with equal quality, respectively.

Magnetic beads were used to enrich the mRNA of eukaryotes by using Oligo-dT [30]. Illumina HiSeq/MiSeq sequencing was then conducted on the cDNA library [31, 32]. The original sequence was sequenced, and sequences containing joints, an uncertain base ratio greater than 10%, and low quality were eliminated. All these procedures were performed by Novogene Co., Ltd. (Beijing, China). Additionally, all reads of transcriptome sequencing have been uploaded to the Sequence Read Archive of the National Center for Biotechnology Information (NCBI, SRA accession number PRJNA1122990).

### 2.3 Analysis of illumina transcriptome sequencing results

A blast search of transcriptomes was performed on 7 databases: NR, NT, KO, Swiss-Prot, PFAM, GO, and KOG. Gene function was categorized using GO and KOG analysis. Subsequently, the sequence was annotated by pathway through KEGG analysis. This helped in analyzing both the metabolic pathway of gene products and compounds in cells and the function of these gene products. The gene expression levels were normalized into RPKM values [33]. p-values and q-values were calculated to identify differentially expressed genes (DEGs). To eliminate biological variation, a threshold was set: | log2 (fold change) | > 1 and q-value < 0.005. This helped in identifying significant differences between the two levels of assessment and the difference in gene screening.

### 2.4 Functional annotation and pathway prediction of DEGs

The male and female DEGs were analyzed using GO annotation and Pathway analysis to better understand the differences in gene function between the sexes in *F*. *mandshurica*. GO analysis allowed us to examine the distribution of differential genes in the Gene Ontology (http://www.geneontology.org/) and identify significantly enriched GO terms using the Goseq method with a p-value threshold of ≤0.05. This provided insight into the major biological functions of DEGs. Moreover, The important biological pathways involved in DEGs through significant pathway enrichment analysis of KEGG (http://www.genome.jp/kegg/) were identified [34].

### 2.5 Primer design and qRT-RCR validation

According to the transcriptome analysis, the genes that need further verification were selected out. Use Primer 5.0 software to design fluorescent quantitative primers (S3 Table), the product length was between 100–300 bp, and the annealing temperature was limited to 55–65°C. The primer was synthesized by Sangon Biotech Co., Ltd. (Shanghai, China). RNA was reverse transcribed into cDNA using the PrimeScript™ RT reagent Kit with gDNA Eraser (TaKaRa, Tokyo, Japan) according to the kit instructions [35].

Real-time quantitative PCR was conducted on a 96-well plate and SYBR® Premix Ex Taq™ II (Tli RNaseH Plus) kit using the Applied Biosystems 7500 real-time PCR system. The 20 μL reaction mixture included 1 μL cDNA (< 100ng), 0.8 μL each of upstream and downstream primers, 10 μL SYBR® Premix Ex TaqTM II, and 0.4 μL Rox Reference Dye II. The PCR procedure involved the following steps: 95°C for 30 seconds, 95°C for 5 seconds, and 60°C for 34 seconds, which was repeated for 40 cycles. The 2^−ΔΔCt^ method was used to calculate the relative gene expression and the internal reference gene was tubulin. All qRT-PCR reactions were performed with the three biological replicates.

### 2.6 Statistical analysis

Data analysis was conducted using SPSS 27 (SPSS, Inc., Chicago, IL, USA), t-test was used to determine the statistical significance (*p < 0.05, **p < 0.01, ***p< 0.001). Furthermore, image generation were performed using Origin 2021 (OriginLab, Northampton, MA, USA) and Excel 2019 (Microsoft, Redmond, WA, USA).

## 3. Results

### 3.1 Transcriptome sequencing data results

**Fig 1:**
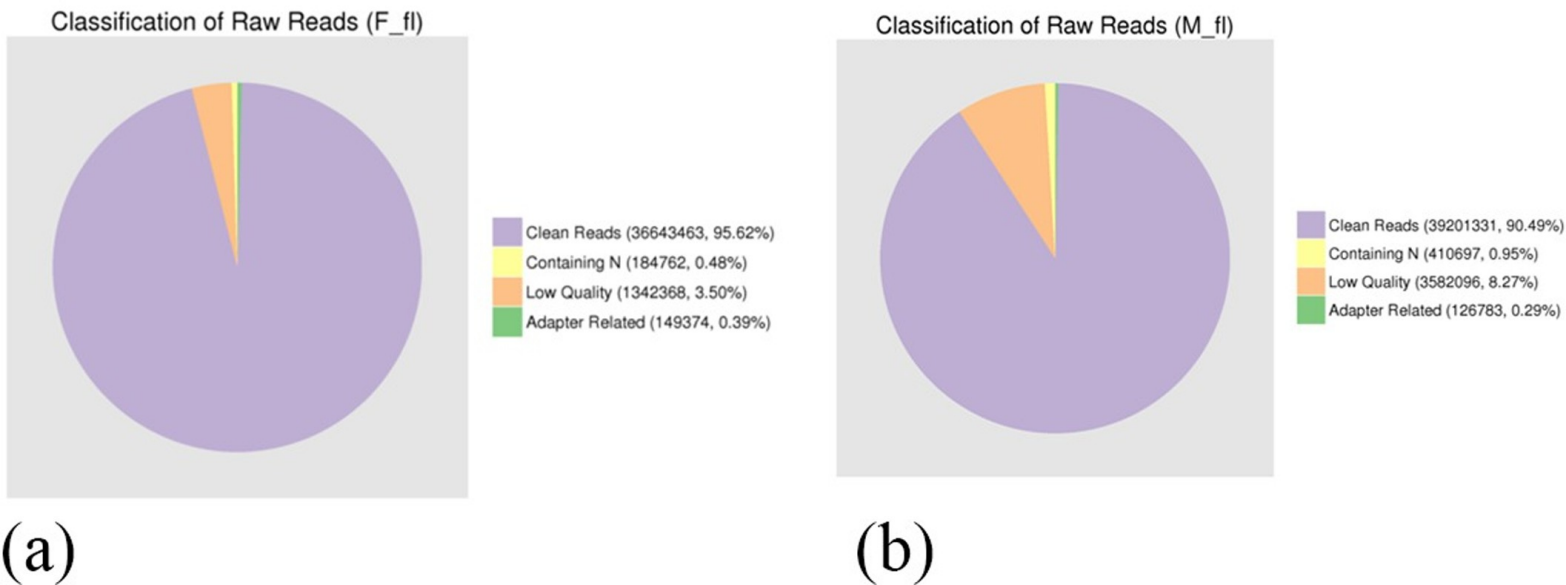
The composition of raw sequence data. (a) The composition of female flower data. (b) The composition of male flower data.

Through transcriptome sequencing, the female and male flower databases generated 38,319,967 and 43,320,907 reads, respectively. Upon screening, it was found that female and male flowers contained 3.66 GB and 3.92 GB of nucleotides, respectively. The error rates of both male and female flower sequences were less than 0.06, with Q20(%) at around 95%, and the GC content at approximately 43% (S1 Table). Moreover, the proportion of clean reads in both samples accounted for more than 90% of raw reads, which proves the higher sequencing quality for both samples. Therefore, the number of clean reads obtained could meet the experiment’s requirements (Fig 1).

To study the functions of the unigenes and gather information about their characteristics, the assembled Unigene was compared with seven public databases using Blast and Blastx methods. The results were then analyzed and counted. Out of the seven databases, 32,311 NR (35.69%), 14,051 NT (15.52%), 9,084 KO (10.03%), 22,583 SwissPro (24.94%), 22,589 PFAM (24.95%), 25,961 GO (28.67%), and 10,879 KOG (12.01%) had sequences that matched. All Unigene sequences obtained homologous alignment information (Table 1).

**Table 1:** Results of functional annotation.

Database	Number of Unigenes	Percentage (%)
Annotated in NR	32311	35.69
Annotated in NT	14051	15.52
Annotated in KO	9084	10.03
Annotated in SwissProt	22583	24.94
Annotated in PFAM	22589	24.95
Annotated in GO	25961	28.67
Annotated in KOG	10879	12.01
Annotated in all Databases	3864	4.26
Annotated in at least one Database	36405	40.21
Total Unigenes	90532	100

**Fig 2:**
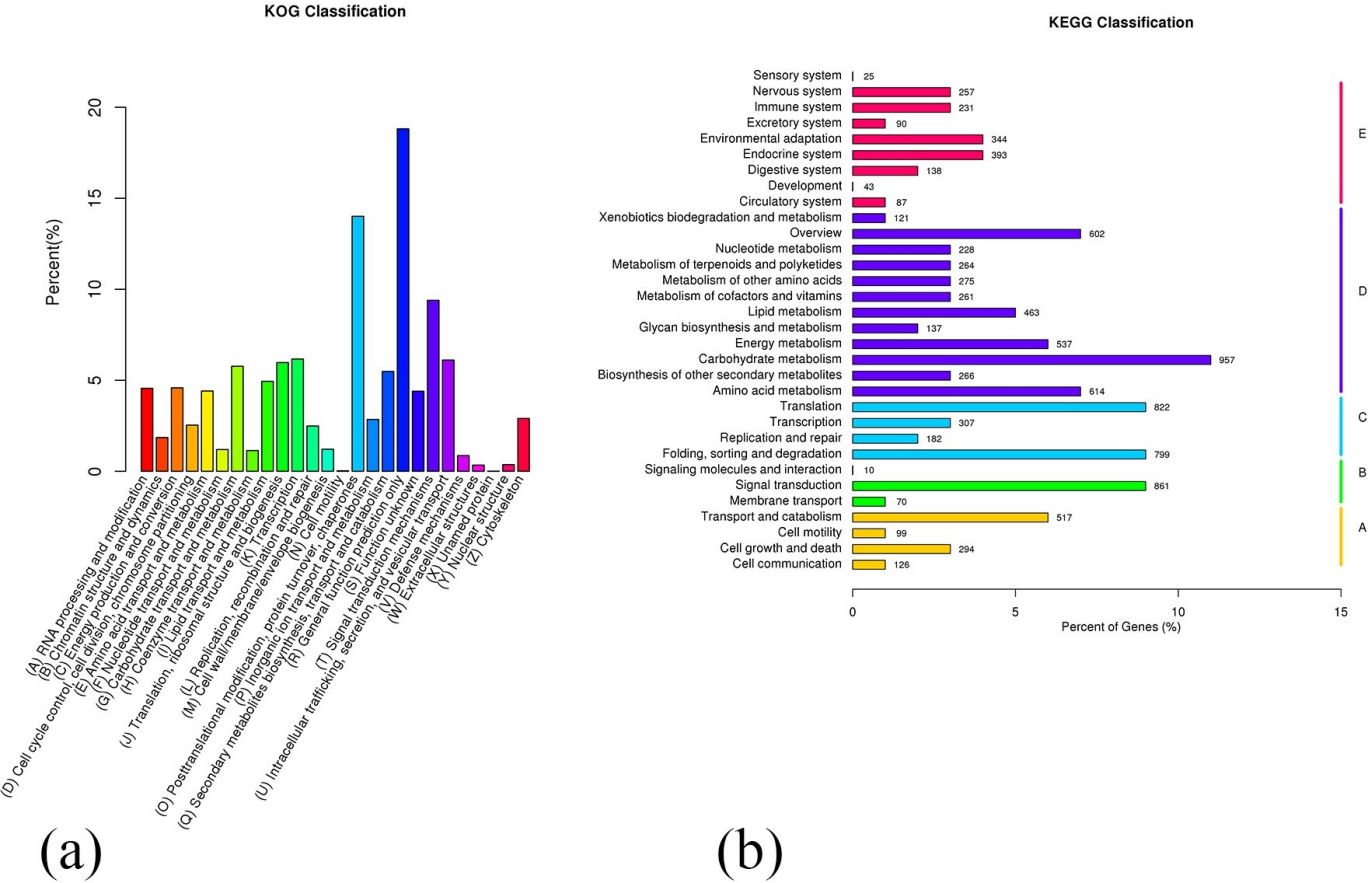
The transcriptome analysis. (a) KOG distribution of transcriptome gene. (b) KEGG classification of transcriptome.

To evaluate the transcriptome annotations’ completeness and validity, the annotated genes was categorized into KOG database. Out of 10,879 sequences, 2,047 genes (16.73%) fell into the General function prediction only (R) category, indicating that there are still many new genes to be developed in *F*. *mandshurica*. Posttranslational modification, protein turnover, and chaperones (O) with 1,524 genes (12.46%), followed by Signal transduction mechanisms (T) with 1,022 genes (8.36%), and Transcription (K) with 671 genes (5.49%). These results suggest that transcription, post-transcriptional processing, and signal transduction play crucial roles in gene expression regulation (Fig 2A). Further analysis using KEGG showed that 9,084 genes are assigned to five major categories, including Metabolism (D), Genetic Information Processing (C), Environmental Information processing (B), Cellular processes (A), and Organismal Systems (E). These genes were involved in 260 KEGG pathways (Fig 2B).

## 3.2 Screening and cluster analysis of DEGs

**Fig 3:**
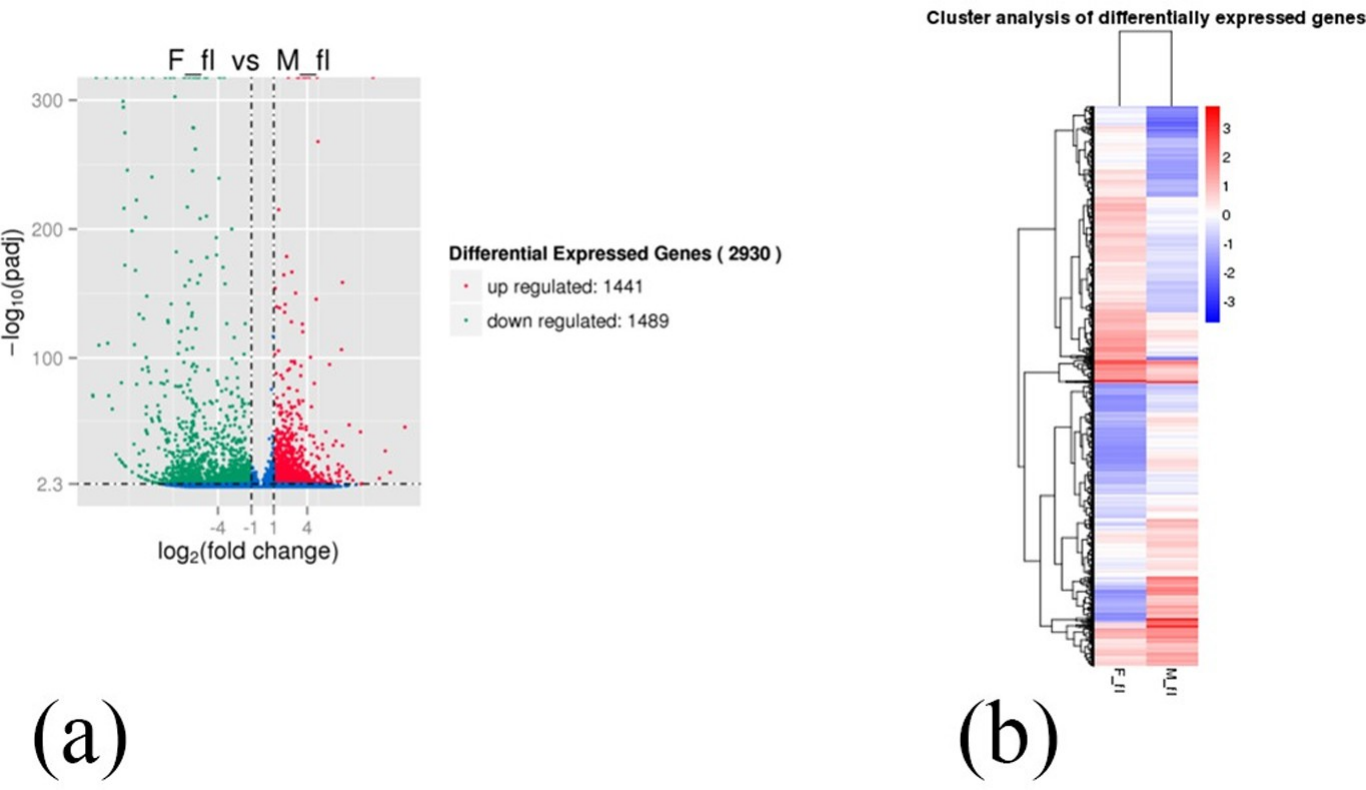
Analysis of DEGs. (a) Volcano map of DEGs. DEGs that may be false positives were shown in blue, up-expressed genes were shown in red, and down-expressed genes were shown in green. (b) Heat map of cluster analysis of DEGs. In the overall RPKM hierarchical clustering graph, red indicated up-regulated genes and blue indicated down-regulated genes.

The expression level of genes in male and female flowers exhibited a similar tendency. The majority of RPKM gene expression ranged from 1 to 15, accounting for 86.25% and 87.25% of the female and male databases, respectively. In *F*. *mandshurica* with RPKM >60, only 3.5% were observed, and the gene expression level followed a normal distribution. The volcano map revealed 2930 DEGs after the screening, with 1,441 genes (49.18%) up-regulated and 1,489 genes (50.82%) down-regulated in females compared to males (Fig 3A). Overall, the DEGs showed a distribution of | log2 (Fold Change) | basic concentration values between 1 ~ 4, indicating a difference ratio of 2 ~ 16 times. A hierarchical clustering analysis was conducted based on RPKM values of the DEGs between males and females, revealing a large number of DEGs in the heat map (Fig 3B).

### 3.3 GO and KEGG analysis of DEGs

**Fig 4:**
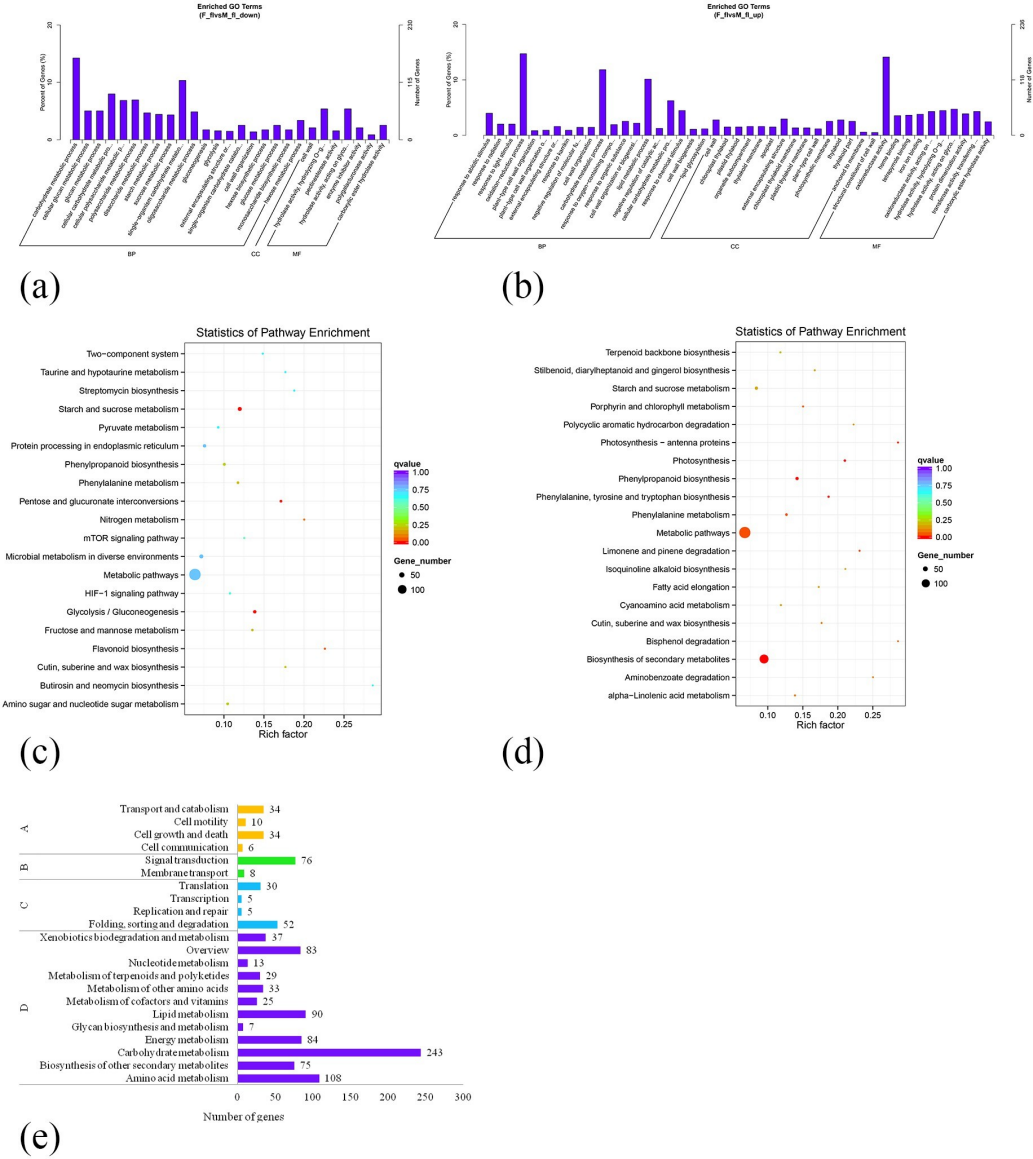
GO and KEGG analysis of DEGs. (a) GO analysis of down-regulated genes. (b) GO analysis of up-regulated genes. (c) KEGG analysis of down-regulated genes. (d) KEGG analysis of up-regulated genes. (e) KEGG classification of DEGs. A: Cellular Processes, B: Environmental Information Processing, C: Genetic Information Processing, D: Metabolism.

To facilitate a more intuitive understanding of gene expression differences, GO analysis was conducted on the up-regulated and down-regulated DEGs separately (Fig 4A and 4B). The differential genes were classified into three functional classes: biological processes (2,081 GO functional annotations, 59.27%, BP), cell components (406 GO functional annotations, 11.6%, CC), and molecular functions (1,024 GO functional annotations, 29.17%, MF). Among the up-regulated genes, significant enrichment was observed in oxidation-reduction processes (BP), lipid metabolic processes (BP), and oxidoreductase activity (MF). Similarly, among the down-regulated genes, there was significant enrichment in carbohydrate metabolic processes (BP) and single-organism carbohydrate metabolic processes (BP). The GO functional annotation analysis showed a large level of genetic divergence in biological processes.

1,612 DEGs were discovered by KEGG Pathway analysis, which was mapped to 151 pathways. The pathways for starch and sucrose metabolism, as well as glycolysis/gluconeogenesis, were significantly down-regulated. Conversely, metabolic pathways and biosynthesis of secondary metabolites were significantly up-regulated (Fig 4C and 4D). Based on biological functions, the DEGs can be classified into 4 categories-metabolism, genetic information processing, environmental information processing, and cellular processes and 22 sub-categories, including transport and metabolism, signal transduction, folding, sequencing, and degradation, as well as carbohydrate metabolism, among others. These indicates that the development of male and female *F*. *mandshurica* involves complex physiological and biochemical processes (Fig 4E).

### 3.4 Analysis of DEGs in the pathways

Out of the 151 pathways of differential genes analyzed, it was found that 59 pathways had more genes expressed in down-expressed, while 10 pathways had equal amounts of genes expressed by both expression. The remaining 82 pathways showed more genes expressed in up-expressed. In terms of female and male flowers development, the plant hormone signal transduction pathway had up to 20 up-expressed genes, while the other three pathways: the flavonoid biosynthesis pathway, the energy metabolism pathway (carbon metabolism and nitrogen metabolism), and the photoperiod pathway had more down-expressed genes (Table 2). Among the above four pathways related to flower development, genes with large multiples of difference were screened out (S2 Table). And 16 genes among these genes were selected for primer design and quantitative analysis (S3 Table).

**Table 2:** Analysis of differentially expressed genes in the pathways.

Pathway	Differential genes	down-expressed genes	up-expressed genes	Difference magnitude
Hormone signal transduction	26	20	6	0.231
Nitrogen metabolism	12	5	7	0.583
Carbon metabolism	32	15	17	0.531
Flavonoid biosynthesis	9	2	7	0.778
Photoperiod	4	1	3	0.750

Note: Difference magnitude = up-regulated genes/total differential genes in a pathway.

### 3.5 The DEGs were verified by qRT-PCR

**Fig 5:**
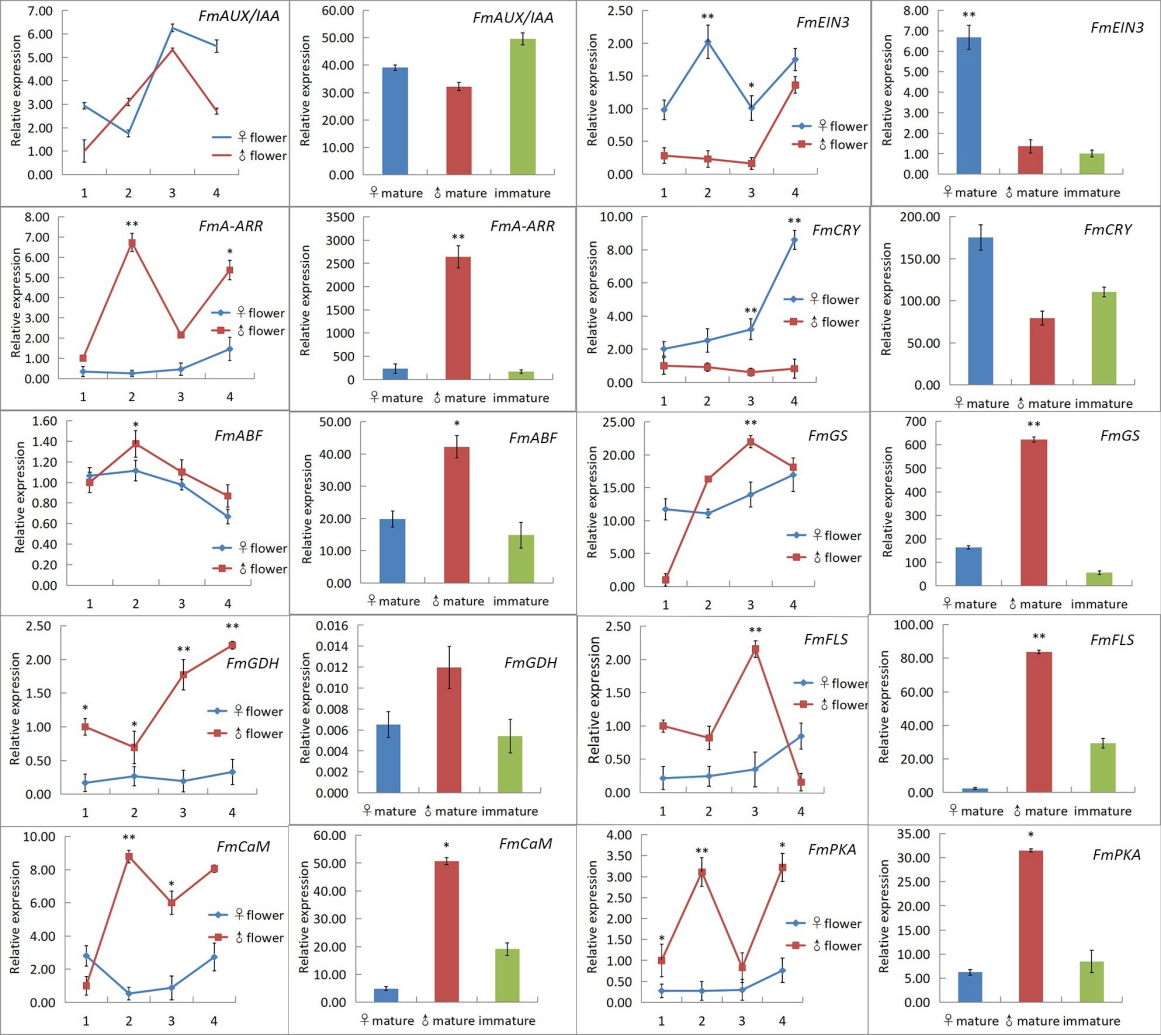
10 genes that were consistently and significantly expressed in flowers and leaves of *F*. *mandshurica*. In the two graphs of the same gene, the left line graph shows expression in flowers, and the right bar graph shows expression in leaves. 1, 2, 3, and 4 represent the four stages of flower development, respectively, ♂1: archesporial cell phase, ♂2: pollen mother cell phase, ♂3: meiosis cell, and ♂4: cell of loose pollen phase. ♀1: archesporial cell phase, ♀2: embryo-sac mother cell phase, ♀3: meiosis cell and ♀4: binucleated cell. The left side of the same gene represents gene expression in the leaves of adult females, adult males, and young trees. Blue represents females, red represents males, and brown represents seedlings. A p-value ≤0.05 was considered significant (*), and a p-value ≤0.01 was considered highly significant (**).

Fluorescence quantitative analysis was conducted on 16 genes in the flowers and leaves of male and female *F*. *mandshurica* plants over four stages. Based on the consistency and significance results of fluorescence quantitative analysis of flowers and leaves of *F*. *mandshurica*, 10 significantly expressed genes were obtained (Fig 5). There was hormone signal transduction (*FmAUX/IAA*, *FmA-ARR*, *FmEIN3*, and *FmABF*), energy metabolism (*FmGS* and *FmGDH*), flavonoid biosynthesis (*FmFLS*), and photoperiod (*FmCaM*, *FmPKA*, and *FmCRY*). The expression of these genes was significantly different between male and female plants, that *FmAUX/IAA*, *FmEIN3*, and *FmCRY* genes were significantly expressed in females, and *FmA-ARR*, *FmABF*, *FmGS*, *FmGDH*, *FmFLS*, *FmCaM*, and *FmPKA* genes were significantly expressed in males. These studies indicate that the transcriptome sequencing data of male and female flowers of *F*. *mandshurica* are appropriate and accurate, and the flower development process is complex, involving multiple pathways and genes.

## 4. Discussion

The use of transcriptome or expression profiles for screening DEGs has become widespread [36–38]. However, transcriptome analysis of flowers of the diecious plant *F*. *mandshurica* has not been reported. Our study can provide a reference for further research on the developmental pathways and gene expression of female and male flowers of *F*. *mandshurica*. In one study, Maria et al. analyzed the gene expression of female and male gametophytes of *Fucus vesiculosus* brown alga and identified 28 female and 92 male over-expressed genes [39]. In another study, He et al. conducted transcriptome analysis on the leaves of *F*. *mandshurica* and hybrid seedlings, revealing 11,321 DEGs in the leaf transcriptome of 1601 and M8 with 6,008 genes up-regulated, and 5,313 down-regulated [40]. In this study, a total of 2,930 DEGs were identified from the transcriptome with 1,441 genes up-regulated, and 1,489 genes down-regulated. The transcriptome analysis provided sufficient DEGs for further analysis and predicted four pathways of gender-related: plant hormone signal transduction, energy metabolism, flavonoid biosynthesis, and photoperiod pathways.

Plant hormones play a significant role in the regulation of sexual development and flowering in plants [41, 42]. Among these hormones, auxin and ethylene are considered to be the primary phytohormones associated with sex development [43–45]. Specifically, auxin is crucial to the development of female plants in hemp [46] and cactus [47], and its signal is up-regulated during the transformation of *Ricinus communis* pistil from leaf tip to flower bud [48]. Ethylene, has a strong effect on female development in cucumbers [49, 50] and melon [51]. Interestingly, female flowers were even found on the male plants of mulberry under a certain concentration of ethylene [52]. This effect is due to the transmission of the ethylene signal, which promotes the expression of the *EIN3* gene and the development of female flowers. In this study, it was found that *FmAUX/IAA* and *FmEIN3* genes were preferentially expressed in females, indicating that the auxin and ethylene signal transduction pathways were stronger in females and promoted their development.

Studies have shown that cytokines (CTK) can have an impact on the gender expression of plants [53], with some evidence suggesting that it could promote female development in *Mercurialis annua* [54]. Furthermore, CTK treatment of male flowers in hemp, grape, and spinach plants has been shown to lead to the transformation of male flowers into female flowers [55]. Abscisic acid (ABA) is known to play a role in regulating various aspects of plant growth and development, including flower bud differentiation, seed formation, and responses to environmental stresses. Sladky’s study found that a certain level of ABA is necessary for the differentiation of female flower buds, but excessive accumulation can halt the process [56]. This study found that the expression of *FmA-ARR* and *FmABF* genes was prominent in males, and the negative feedback mechanism of *A-ARR* regulating *B-ARR* inhibiting its expression was the reason for the significant expression of *A-ARR* genes. These findings suggest that cytokinin can stimulate the production and development of female flowers, while abscisic acid is important for the development and stress resistance of male flowers. More information about the regulatory networks of plant hormone signal transduction pathway is available at KEGG website (https://www.kegg.jp/kegg-bin/show_pathway?ko04075).

The development trends in energy metabolism and flavonoid biosynthesis differ between male and female plants. When nutrients are limited, female plants prioritize nutrient acquisition, while male plants allocate more resources to above-ground growth [57]. Flavonoids have been found to protect plant DNA from UV damage [58], and UV-stressed Arabidopsis plants have been observed to have higher flavonoid content [59]. Additionally, flavonoids have important effects on plant reproductive activities, promoting functional pollen tube formation and pollen germination [60]. In plants, FLS catalyzes dihydroquercetin to form flavonols such as kaempferol, quercetin and myricetin [61]. In cells, NH_4_^+^ is assimilated into glutamine under the action of glutamine synthetase (GS), which uses ATP to provide energy. Glutamate synthase (GOGAT) catalyzes the formation of glutamate from glutamine [62]. In this study, *FmGS* and *FmGDH* genes of energy metabolism pathway and *FmFLS* genes of flavonoid biosynthesis pathway were significantly expressed in male flowers of *F*. *mandshurica*. This suggests that male plants have a higher level of nitrogen metabolism and flavonoid biosynthesis, potentially contributing to their rapid growth and pollen formation.

The length of daylight, or photoperiod, has a notable impact on the flower development in plants [63]. In a study of *Arabidopsis thaliana*, researchers discovered that longer days encouraged flowering, while shorter days inhibited it [64]. The photoperiod pathway primarily detects and reacts to light signals through photoreceptors and circadian clocks to stimulate the transition to flowering [65]. In a gene chip screening of DEGs between male and female *Populus tomentosa*, researchers found that *PIL5* expression was notably increased in male flowers [66]. This study found that *FmCRY* gene was prominently expressed in females, which consequently hindered the function of the Clock-Bmal1 heterodimer and reduced the translation of Cry and period genes. Calcium and Ca^2+^ signaling plays a crucial role in flagellar and sperm motility, and given that *FmCaM* and *FmPKA* genes were highly expressed in males, it is believed that this dominant expression of photoperiod-related genes in male flowers may be linked to their more active biological activities.

## 5. Conclusions

In this study, we analyzed the transcriptome sequencing data of male and female flowers at four different developmental stages of *F*. *mandshurica*. 2930 DEGs were yielded, with 1441 genes in up-regulated and 1489 genes in down-regulated. Through GO and KEGG analysis, DEGs were enriched in four pathways related to flower development. Further validation through qRT-PCR demonstrated that 10 genes in these pathways showed significant differences, aligning with the transcriptome analysis. There were *FmAUX/IAA*, *FmA-ARR*, *FmEIN3*, *FmABF* genes in hormone signal transduction; *FmGS* and *FmGDH* genes in energy metabolism; *FmFLS* gene in flavonoid biosynthesis; and *FmCaM*, *FmPKA*, and *FmCRY* genes in photoperiod. Our findings offer valuable insights into the differential pathways and gene expression patterns of male and female flowers of *F*. *mandshurica* during development and serve as a reference for further studies on plant sex development.

## Supporting information

S1 Fig RNA quality testing for *F*. *mandshurica*. (DOCX)

S1 Table Summary of the *F*. *mandshurica* transcriptome. (DOCX)

S2 Table Flower development related pathways and genes. (DOCX)

S3 Table Genes and primer sequences of qRT-PCR. (DOCX)
