# Supplementary material for: Comparative transcriptome analysis of differentially expressed genes and pathways in male and female flowers of Fraxinus mandshurica
Source: PLoS One. 2024 Sep 12;19(9):e0308013. doi: 10.1371/journal.pone.0308013 (PMC11392328; doi:10.1371/journal.pone.0308013)
Supplement: S1 Fig — (DOCX) [file pone.0308013.s001.docx]

**S1 Fig. RNA quality testing for *F. mandshurica*.**

The quality of RNA is detected by electrophoresis, 28s:18s≈2:1, RNA is intact without degradation, without DNA and protein contamination. UV detection of RNA showed that the A260:A280 values were between 1.8 and 2.0; the concentrations were all greater than 500μg/ml, which met the experimental requirements; and a smooth peak appeared at A260nm, indicating that the RNA was free of degradation and contamination (S1 Fig).

**S1
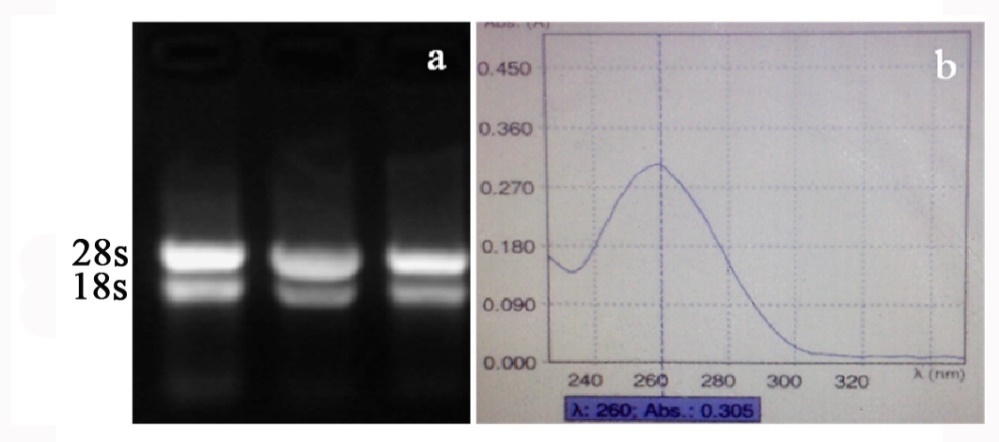
 Fig. Results of total RNA.** a. Results of 1.0% agarose gel electrophoresis. b. Result of UV detection.
