# Supplementary material for: Comparative transcriptome analysis of differentially expressed genes and pathways in male and female flowers of Fraxinus mandshurica
Source: PLoS One. 2024 Sep 12;19(9):e0308013. doi: 10.1371/journal.pone.0308013 (PMC11392328; doi:10.1371/journal.pone.0308013)
Supplement: S1 Table — (DOCX) [file pone.0308013.s002.docx]

**S1 Table. Summary for the *F. mandshurica* transcriptome.**

| **Sample** | **Raw Reads** | **Clean Reads** | **Clean Bases** | **Error(%)** | **Q20(%)** | **GC Content(%)** |
| --- | --- | --- | --- | --- | --- | --- |
| F_fl_1 | 38319967 | 36643463 | 3.66G | 0.05 | 95.59 | 43.15 |
| F_fl_2 | 38319967 | 36643463 | 3.66G | 0.06 | 94.33 | 43.21 |
| M_fl_1 | 43320907 | 39201331 | 3.92G | 0.03 | 97.76 | 43.05 |
| M_fl_2 | 43320907 | 39201331 | 3.92G | 0.06 | 93.82 | 43.08 |

Note: Q20 (%) refers to the proportion of reads whose average cycle value is greater than or equal to 20.
