# Supplementary material for: Comparative transcriptome analysis of differentially expressed genes and pathways in male and female flowers of Fraxinus mandshurica
Source: PLoS One. 2024 Sep 12;19(9):e0308013. doi: 10.1371/journal.pone.0308013 (PMC11392328; doi:10.1371/journal.pone.0308013)
Supplement: S2 Table — (DOCX) [file pone.0308013.s003.docx]

**S2 Table. Flower development related pathways and genes.**

| **Pathway** | **Gene Name** | **log2.Fold_change** | **Description** | |
| --- | --- | --- | --- | --- |
| Hormone signal transduction | *FmAUX1* | 6.8753 | auxin influx carrier (AUX1 LAX family) | |
|  | *FmAUX/IAA* | 3.1872 | auxin-responsive protein IAA | |
|  | *FmGH3* | 2.7039 | auxin responsive GH3 gene family | |
|  | *FmSAUR* | -5.6431 | SAUR family protein | |
|  | *FmAHP* | 1.0381 | histidine-containing phosphotransfer peotein | |
|  | *FmA-ARR* | -4.6514 | two-component response regulator ARR-A family | |
|  | *FmDELLA* | 3.4979 | DELLA protein | |
|  | *FmPP2C* | 4.7967 | protein phosphatase 2C | |
|  | *FmSNRK2* | 1.3022 | serine/threonine-protein kinase SRK2 | |
|  | *FmABF* | -9.0885 | ABA responsive element binding factor | |
|  | *FmEIN3* | 1.1158 | ethylene-insensitive protein 3 | |
|  | *FmBSK* | 1.0938 | BR-signaling kinase | |
|  | *FmCYCD3* | 2.1675 | cyclin D3, plant | |
|  | *FmCOI-1* | -3.7602 | coronatine-insensitive protein 1 | |
|  | *FmJAZ* | 6.0167 | jasmonate ZIM domain-containing protein | |
|  | *FmMYC2* | 2.4089 | transcription factor MYC2 | |
|  | *FmTGA* | -6.373 | transcription factor TGA | |
|  | *FmGID1* | 1.3244 | gibberellin receptor GID1 | |
| Nitrogen assimilation | *E3.5.1.49* | 1.5585 | formamidase | |
|  | *FmcynT* | -12.8527 | carbonic anhydrase | |
|  | *FmGDH* | -3.0458 | glutamate dehydrogenase (NAD(P)+) | |
|  | *FmGS* | -7.4091 | glutamine synthetase | |
| Carbon metabolism | *FmHK* | -5.4927 | hexokinase |  |
|  | *Fm6PGDH* | -1.0908 | 6-phosphogluconate dehydrogenase | |
|  | *FmENO* | -3.0937 | enolase |  |
|  | *FmPK* | -2.2048 | pyruvate kinase | |
|  | *FmACSS* | -1.0663 | acetyl-CoA synthetase | |
|  | *FmMDH* | -4.8762 | malate dehydrogenase | |
|  | *FmPGK* | -1.0601 | phosphoglycerate kinase | |
|  | *FmG6PD* | 1.6086 | glucose-6-phosphate 1-dehydrogenase | |
|  | *Fmtkt* | 1.3695 | transketolase | |
|  | *FmfdhA* | 4.4617 | glutathione-independent formaldehyde dehydrogenase | |
|  | *E3.5.1.49* | 1.5585 | formamidase | |
| Flavonoid biosynthesis | *FmCHS* | -3.2115 | chalcone synthase | |
|  | *FmCYP73A* | -2.5946 | trans-cinnamate 4-monooxygenase | |
|  | *E1.14.11.9* | -2.7358 | naringenin 3-dioxygenase | |
|  | *FmFLS* | -3.3668 | flavonol synthase | |
|  | *E1.14.13.21* | -1.0961 | flavonoid 3'-monooxygenase | |
|  | *E2.1.1.104* | 8.1777 | caffeoyl-CoA O-methyltransferase | |
|  | *FmCYP98A3* | 2.4499 | coumaroylquinate(coumaroylshikimate) 3'-monooxygenase | |
|  | *FmLAR* | 6.1946 | leucoanthocyanidin reductase | |
| Photoperiod | *FmCaM* | -2.12 | calmodulin |  |
|  | *FmPKA* | -3.17 | protein kinase A | |
|  | *FmRBX1* | -4.59 | RING-box protein 1 | |
|  | *FmCRY* | 5.1 | cryptochrome | |
