# Supplementary material for: Comparative transcriptome analysis of differentially expressed genes and pathways in male and female flowers of Fraxinus mandshurica
Source: PLoS One. 2024 Sep 12;19(9):e0308013. doi: 10.1371/journal.pone.0308013 (PMC11392328; doi:10.1371/journal.pone.0308013)
Supplement: S3 Table — (DOCX) [file pone.0308013.s004.docx]

**S3 Table.** **Genes and primer sequences of qRT-PCR.**

| **Name of the primer** | **Sequence (5'to3')** |
| --- | --- |
| *FmAUX/IAA-F* | TAAGGTTTACAAGAACTACCCAGAT |
| *FmAUX/IAA-R* | CCCACTAACATCCAATCCC |
| *FmSAUR-F* | TTAGGCAAGAAACAAGGG |
| *FmSAUR-R* | CAGGGTGAGACAAGAAAG |
| *FmGID1-F* | ACGGGTTTGATGTGCTGAAGTT |
| *FmGID1-R* | CGTGATGGGCCAGATTTGCT |
| *FmDELLA-F* | TAGCAGACGCATTGGTGAAGC |
| *FmDELLA-R* | CATTTGAAGAACATCGGAATAGGA |
| *FmAHP-F* | AAGGCTTATCAATGATCTTTCC |
| *FmAHP-R* | TTCACCCTCTGAGCACCA |
| *FmA-ARR-F* | GGTGCTTTGACTTCTGACTGG |
| *FmA-ARR-R* | GCTGTTTACTGGGCTGCTGT |
| *FmEIN3-F* | GGGAAGAATGAGGGGTCCAA |
| *FmEIN3-R* | TGATGCCACCATTCCTCCTT |
| *FmABF-F* | CCGCAGCCTCTACAGCAA |
| *FmABF-R* | ATTCCCAGTCCTCCACCCT |
| *FmGS-F* | GTGCCTGTGGAGGAATCAAG |
| *FmGS-R* | TCAGCGAAAGGTGCGTCA |
| *FmGDH-F* | GAACGCCTTACCCGTGTCT |
| *FmGDH-R* | GCCTCCCTGCCTAGTGAACC |
| *FmPGK-F* | CGACAGAGGGAGTGACCAAG |
| *FmPGK-R* | TCATTCCTCCACCCAATAAGA |
| *FmFLS-F* | GCCAAAGAACCCACCCTC |
| *FmFLS-R* | TTTCATTCCCTTCCAGTCCA |
| *FmCaM-F* | TAGCCTATTCGACAAAGACGG |
| *FmCaM-R* | CATTACCATCGGCATCAACC |
| *FmPKA-F* | TCTAAGGGCATAATGCACAGG |
| *FmPKA-R* | CCAACACTCCACCAATCAGC |
| *FmCRY-F* | TTCCCGATGGTCGTGAGT |
| *FmCRY-R* | GCTTCTTGTAACCTGGCTTTT |
| *FmRBX1-F* | CTGGTGCTGGTCCTTCCTCC |
| *FmRBX1-R* | GAAATTCCCACTCGCTGTTGTC |
| *TU-F* | AGGACGCTGCCAACAACTTT |
| *TU-R* | TTGAGGGGAAGGGTAAATAGTG |
